# Identification of Neural Networks That Contribute to Motion Sickness through Principal Components Analysis of Fos Labeling Induced by Galvanic Vestibular Stimulation

**DOI:** 10.1371/journal.pone.0086730

**Published:** 2014-01-23

**Authors:** Carey D. Balaban, Sarah W. Ogburn, Susan G. Warshafsky, Abdul Ahmed, Bill J. Yates

**Affiliations:** 1 Department of Otolaryngology, University of Pittsburgh, Pittsburgh, Pennsylvania, United States of America; 2 Department of Bioengineering, University of Pittsburgh, Pittsburgh, Pennsylvania, United States of America; 3 Department of Communication Sciences and Disorders, University of Pittsburgh, Pittsburgh, Pennsylvania, United States of America; 4 Department of Neurobiology, University of Pittsburgh, Pittsburgh, Pennsylvania, United States of America; 5 Department of Neuroscience, University of Pittsburgh, Pittsburgh, Pennsylvania, United States of America; University of Medicine & Dentistry of NJ - New Jersey Medical School, United States of America

## Abstract

Motion sickness is a complex condition that includes both overt signs (e.g., vomiting) and more covert symptoms (e.g., anxiety and foreboding). The neural pathways that mediate these signs and symptoms are yet to identified. This study mapped the distribution of c-fos protein (Fos)-like immunoreactivity elicited during a galvanic vestibular stimulation paradigm that is known to induce motion sickness in felines. A principal components analysis was used to identify networks of neurons activated during this stimulus paradigm from functional correlations between Fos labeling in different nuclei. This analysis identified five principal components (neural networks) that accounted for greater than 95% of the variance in Fos labeling. Two of the components were correlated with the severity of motion sickness symptoms, and likely participated in generating the overt signs of the condition. One of these networks included neurons in locus coeruleus, medial, inferior and lateral vestibular nuclei, lateral nucleus tractus solitarius, medial parabrachial nucleus and periaqueductal gray. The second included neurons in the superior vestibular nucleus, precerebellar nuclei, periaqueductal gray, and parabrachial nuclei, with weaker associations of raphe nuclei. Three additional components (networks) were also identified that were not correlated with the severity of motion sickness symptoms. These networks likely mediated the covert aspects of motion sickness, such as affective components. The identification of five statistically independent component networks associated with the development of motion sickness provides an opportunity to consider, in network activation dimensions, the complex progression of signs and symptoms that are precipitated in provocative environments. Similar methodology can be used to parse the neural networks that mediate other complex responses to environmental stimuli.

## Introduction

Vomiting is usually considered to be a protective reflex to rid the body of ingested toxins. However, this response is also elicited following surgery or exposure to radiation, during cancer chemotherapy or pregnancy, and even as a consequence of some psychological stimuli [1], [2]. Vestibular stimulation can also result in emesis, particularly during conditions where sensory inputs provide contradictory information regarding body position in space [3], [4]. It is generally assumed that emesis, despite its triggering mechanism, is mediated through a “final common pathway” [4]–[8]. The same output pathways that produce vomiting in response to toxins are thus also presumably involved in generating motion sickness-related emesis. One strong piece of evidence to support the final common pathway hypothesis is the existence of broad-spectrum antiemetics, such as neurokinin-1 (NK_1_) receptor antagonists, that prevent vomiting despite the provocation [9]–[15]. NK_1_ receptor antagonists are effective in a variety of species, including humans, musk shrews, ferrets, dogs, and cats, suggesting that the neural pathways that produce vomiting are similar across emetic animals. However, many animals, including the most commonly used species in biomedical research (rodents and rabbits), lack the capacity to vomit [16]. This is due both to reduced muscularity of the diaphragm and a stomach geometry that is not well structured for moving contents towards the esophagus in non-emetic animals. In addition, the brainstem circuitry that regulates the respiratory muscle contractions that result in vomiting differs between emetic and non-emetic animals [16], [17].

The sensation of nausea usually precedes vomiting, and is complex [4], as it includes epigastric awareness and discomfort along with anxiety and foreboding regarding the emesis that could ensue [18]–[20]. Some of the symptoms that occur during motion sickness, such as pallor and cold sweating, have been associated with the stress accompanying the condition [5]. Studies conducted in a variety of animal species have attempted to determine the brain regions that mediate nausea and vomiting by mapping the distribution of c-fos protein (Fos)-like immunoreactivity elicited during this behavior [21]–[28]. c-fos is an immediate-early gene that is rapidly expressed in response to neuronal activation. After being synthesized in the cytoplasm, Fos is quickly translocated to the nucleus where, with the Jun protein, it forms a heterodimer that regulates the expression of other genes [29], [30]. As such, Fos expression indicates that a neuron is activated during a particular response. However, most previous experiments that included emetic stimuli only considered Fos distribution in a restricted region of the brainstem such as nucleus tractus solitarius (NTS) [26], [28], [31] or circumscribed areas of the caudal medulla [21], [23], [24], [27]. Furthermore, only two studies have mapped Fos expression during motion sickness [23], [24]. Both studies were conducted on shrews, which were placed on a tabletop shaker to stimulate the vestibular system, and limited the mapping of Fos to NTS and the adjacent reticular formation.

The goal of the present experiment was to perform a comprehensive analysis of the distribution of Fos immunoreactivity during vestibular-elicited nausea and emesis. Felines were used as the model animal during these studies, since most neurophysiological experiments probing the neural mechanisms that produce vomiting have been conducted in cats [2], [27], [32]–[53]. The cat is a species for which there is extensive background information regarding the vestibular and respiratory control systems [54]–[58]. There are limited data about either the vestibular system or respiratory regulation in other emetic animals, including dogs, shrews and ferrets. Although nonhuman primates have been extensively employed in studies of the vestibular system, little work in these animals has addressed the neural mechanisms that regulate respiratory muscle contractions. Consequently, cats were the most appropriate emetic animals to be employed in these experiments.

To evoke motion sickness, 90° out-of-phase galvanic stimulation of the two labyrinths was delivered [59], [60]. Prior studies have shown that this regimen effectively produces emesis and related prodromal symptoms in a subset of animals by generating a novel pattern of vestibular inputs. However, unlike the complex motion stimuli required to evoke motion sickness [61]–[78], this method does not stimulate nonlabyrinthine receptors [59], [60]. Thus, use of galvanic electrical stimulation in these experiments discounted the possibility that Fos labeling was related to stimulation of receptors whose inputs were unrelated to the generation of motion sickness, such as those activated by fluid shifts in the body. Due to the possibility that different animals might have distinct responses during stimulation, with some focused on the stress component of motion sickness and others focused on epigastric awareness and discomfort, we also considered whether the pattern of Fos immunoreactivity was matched to the particular signs and symptoms that each animal expressed. Furthermore, our initial studies indicated that brainstem regions containing serotoninergic neurons exhibited high levels of Fos immunoreactivity during galvanic vestibular stimulation, so we additionally performed dual-labeling immunohistochemistry to detect Fos and tryptophan hydroxylase-2 (TPH2), the brain-specific isoform of the enzyme responsible for the initial and rate-limiting step in serotonin synthesis [79].

## Materials and Methods

All experimental procedures conformed to the National Research Council *Guide for the Care and Use of Laboratory Animals* and were approved by the University of Pittsburgh’s Institutional Animal Care and Use Committee. Data were collected from 10 purpose-bred adult cats (Liberty Research, Waverly, NY) of either sex, weighing 2.4 to 4.6 kg at the conclusion of the experiment. Information about the animals is provided in Table 1.

**Table 1:** Characteristics about the animals used in these experiments, as well as the maximal voltage delivered to the labyrinths to induce motion sickness and the period of acclimation to experimental conditions prior to the stimulation session.

Animal Number	Maximum Stimulation Voltage	Acclimation Period *(days)*	Weight at Perfusion *(kg)*	Gender
C39	3 V	31	4.6	Male
C52	4 V	34	4.1	Female
C15	3 V	56	4.0	Male
C62	3 V	29	2.4	Female
C64	5 V	44	3.3	Female
C02	5 V	64	2.5	Female
C20	5 V	67	4.0	Male
C41	5 V	30	4.2	Male
C83	0 V	58	4.0	Male
C84	0 V	66	3.8	Male

Animals were brought to the testing room daily for 29–66 days (median of 50 days), for acclimation to the environment and the investigators. During this period, animals were also gradually conditioned for 90 min of restraint in a vinyl bag that encompassed the limbs and torso. We found that animals adapted more readily to restraint when held by an investigator during the experimental session. The same individual handled a particular animal during the course of the experiment, from initial acclimation through the final session when labyrinthine stimulation and then euthanasia were performed. During each session, the laboratory was dark and quiet to avoid startling the animal. Following daily restraint periods, animals were allowed to play in the testing room and were offered a food reward, to further reinforce that the environment was not threatening.

### Surgical Procedures

Midway through the acclimation period, an aseptic surgical procedure was performed in a dedicated operating suite to implant stimulating electrodes adjacent to the labyrinth on each side. During the surgery, animals were initially anesthetized using an intramuscular injection of ketamine (20 mg/kg) and acepromazine (0.2 mg/kg), an endotracheal tube was inserted, and anesthesia was maintained using 1–2% isoflurane vaporized in O_2_. The tympanic bulla was opened using a ventrolateral approach. A silver ball electrode with a tip diameter of ∼0.6 mm was secured using dental cement to the round window, and a second electrode was attached <5 mm away, adjacent to the promontory of the tympanic cavity. The electrodes were insulated except at the tip and attached to Cooner wire, which was led underneath the skin to the top of the head and soldered to a connector. The connector was subsequently attached to the skull using dental cement. After this surgery, animals received antibiotics (amoxicillin, two 50-mg oral doses per day) for 10 days, and analgesia (fentanyl transdermal system, 25 µg/h; Janssen Pharmaceutical Products, Titusville, NJ) for 72 h. The final stimulation session occurred 7–41 days (median of 19 days) following the surgical implantation of electrodes.

### Labyrinthine Stimulation and Euthanasia

During acclimation periods after the implantation of electrodes, the stimulator was attached to the head-mounted connector via a cable, although no voltage was delivered, to mimic conditions during the final stimulation session. In the final session, 0.5 Hz sinusoidal galvanic stimulation was provided through the electrodes to the two labyrinths; the sine waves delivered to the left and right sides were 90° out-of-phase. Voltage sinusoids were generated by a Micro1401 mk 2 data acquisition system controlled by Spike2 version 6 software (Cambridge Electronic Design, Cambridge, UK). We began the session by delivering 1 V stimuli, and the intensity was gradually increased over the next few minutes to a level that generated nystagmus and sinusoidal head movements. The maximal voltage employed was 3 V for two animals, 4 V for one animal, and 5 V for four animals (see Table 1). However, 5 V stimuli elicited little perceptible response in two of the animals (C20 and C41). The other two animals (C83 and C84) served as controls, and were connected to the stimulator but no current was delivered through the electrodes. The finding that symptom severity was not correlated with the stimulus intensities delivered is not surprising. It is established that individuals have varying susceptibility for motion sickness, and that motion sickness symptoms vary between individuals [3]–[5].

Throughout the stimulation period, the animals were carefully monitored, and the presence of the following behaviors was scored: sinusoidal head roll, nystagmus, sinusoidal limb movement (periodically observed by unzipping the restraint bag), licking, retching, salivation, sinusoidal pinna movements, panting, defecation, urination, sedation, vocalization, or thrashing in the restraint bag (interpreted as an attempt to escape the stimulus). If the latter two behaviors persisted for more than a few seconds, the stimulus intensity was decreased until they abated. When scoring behaviors, we noted whether they were overt or just weakly perceptible. A semiquantitative, cumulative behavioral score was also generated for each animal by assigning animals with overt symptoms in a particular category a score of 2, and those with weak symptoms a score of 1. These scores were then added for the 13 behaviors monitored, as indicated in Table 2.

**Table 2:** Behaviors exhibited by animals during galvanic vestibular stimulation: ***A,*** sinusoidal head roll at the frequency of the stimulus; ***B,*** nystagmus; ***C,*** frequent licking; ***D,*** retching; ***E,*** excessive salivation; ***F,*** sinusoidal pinna movement at the frequency of the stimulus; ***G,*** vocalization; ***H,*** panting; ***I,*** thrashing in the restraint bag, presumably as an attempt to escape the stimulus; ***J,*** defecation during the stimulation session; ***K,*** urination during the stimulation session; ***L,*** sinusoidal limb movements at the frequency of the stimulus; ***M,*** sedation (sleeping during the majority of the stimulation session).

Animal	Classification(Score sum)	A	B	C	D	E	F	G	H	I	J	K	L	M
*C39*	*1 (20)*	++	++	++	++		++	++	++	++	++	++		
*C52*	*1 (20)*	++	++	++	++	++	++	++	++	++	+		+	
*C15*	*1 (12)*	++	++	++	++	++			++					
*C62*	*2 (7)*	++	++	++	+									
*C64*	*2 (6)*	++		+		+		++						
*C02*	*2 (5)*	++	++	+										
*C20*	*3 (4)*	+					+							++
*C41*	*3 (3)*	+												++
*C83*	*3 (0)*													
*C84*	*3 (0)*													

The behaviors were graded as either being overt **(++)** or only weakly perceptible **(+)**. Blank cells indicate that the behavior was not present. Based on these behaviors, we classified the stimulus as being highly effective in generating responses (1), moderately effective in generating responses (2), or ineffective (3). The later category includes two animals (C83 and C84) that served as unstimulated controls. A score sum was generated by assigning a score of 2 to overt symptoms (++), and a score of 1 to weak symptoms (+).

After 90 minutes, stimulation was discontinued, and the animals were released from restraint and allowed to ambulate in the laboratory for 60 minutes, as during the acclimation period, but were not provided food or water. This post-stimulation period allowed for the expression of Fos by neurons that were activated during stimulation. The animals with the most profound responses to stimulation typically remained sedentary during the recovery period. Subsequently, animals were anesthetized using ketamine (15 mg/kg) and acepromazine (1 mg/kg) injected intramuscularly, followed by pentobarbital sodium (40 mg/kg) injected intraperitoneally. After verifying the absence of nociceptive reflexes, the animals were perfused transcardially with 1 liter of heparinized saline followed by 2 liters of 4% paraformaldehyde-lysine-periodate fixative [80].

### Tissue Processing

The brainstem was removed, postfixed 1–2 days in 4°C paraformaldehyde-lysine-periodate, and cryoprotected in 30% sucrose in 0.1 M phosphate-buffered saline (PBS) for 2 days. Sections were cut at a thickness of 40 µm using a freezing microtome and collected in six bins of cryoprotectant [81]. Procedures for Fos immunohistochemistry were adapted from a protocol supplied by Dr. Charles Horn [22], [82]. Two wells of sections were rinsed in PBS to remove cryoprotectant, and then a sequence of incubation steps was done in 1% sodium borohydride in PBS, 0.3% hydrogen peroxide in PBS, and 5% normal goat serum (NGS) in PBS containing 0.2% triton X-100 (PBS-TX), with rinses between each step. Subsequently, sections were incubated for 24 h at room temperature with gentle agitation in 1∶5000 rabbit polyclonal anti-Fos antibody (sc-52; Santa Cruz Biotechnology, Santa Cruz, CA) or 1∶1000 mouse monoclonal anti-Fos antibody (sc-8047; Santa Cruz Biotechnology) containing 1% NGS in PBS. Following rinses in PBS, sections were placed in 1∶1000 biotinylated secondary antibody (Jackson ImmunoResearch Laboratories, West Grove, PA) for 3 h at room temperature with gentle agitation. Sections were then rinsed twice in PBS-TX and once in PBS, and then incubated in ABC reagent (Vector Laboratories, Burlingame, CA) for three hours. After rinsing in 0.1 M acetate buffer, sections were placed in 3,3′-diaminobenzidine (5 mg/ml in 175 mM acetate–10 mM imidazole buffer, pH 7.4) with nickel sulfate (25 mg/ml) for 0.5–1 min for the chromogen reaction. Sections were then mounted on gelatin-coated slides, cleared in ascending concentrations of ethanol followed by three changes of xylene, and coverslipped with Cytoseal 60 (VWR Scientific, West Chester, PA) or DPX (Sigma-Aldrich, St Louis, MO). One bin of sections was counterstained with neutral red for identification of cytoarchitecture.

A third bin of tissue from all the cases except the unstimulated controls (animals C83 and C84) was processed for dual localization of Fos and TPH2. As a first step, tissue was processed as described above using mouse monoclonal anti-Fos antibody. After completing the chromagen reaction to visualize Fos as a blue-black reaction product in the cell nuclei, sections were rinsed in PBS, and avidin-biotin immunoperoxidase techniques [83] were used to detect TPH2-containing neurons. A 1∶2000 concentration of rabbit anti-TPH2 antibody (supplied by the laboratory of Dr. Stanley Watson at the University of Michigan) was employed in the analysis; we have previously described the specificity of this antibody in detecting serotoninergic neurons in cats [84]. Subsequently, sections were mounted onto slides and coverslipped as described above.

### Tissue Analysis

Following an initial qualitative analysis, 10 or more sections from each animal were selected for quantitative analysis of the distribution of Fos immunoreactivity. The distance of each section anterior (A) or posterior (P) to stereotaxic zero was determined by reference to Berman’s atlas [85]. The following brainstem levels were included in the quantitative analysis: P16, through the commissural nucleus tractus solitarius (NTS); P13.5, through the obex; P12.5, through the rostral NTS; P9.5, through the inferior vestibular nucleus and caudal aspect of the medial vestibular nucleus; P7.5, through the lateral vestibular nucleus; P5.5, through the superior vestibular nucleus; P4, through the parabrachial nucleus; P2, through the caudal aspect of the inferior colliculus; P1, through the caudal periaqueductal gray; A2, through the rostral periaqueductal gray. Sections were photographed using a 4X objective of a Nikon Eclipse E600N photomicroscope equipped with a Spot RT monochrome digital camera (Diagnostic Instruments, Sterling Heights, MI) and MetaMorph imaging software (Universal, Downingtown, PA). Montages of images were assembled using PTGui-Pro photostitching software (New House Internet Services B–V, The Netherlands). These montages, in conjunction with observations of sections at high magnification, were used to generate counts of labeled cells in target areas, as well as plots of the locations of labeled cells. These plots provided the data utilized in subsequent statistical analyses.

When analyzing sections processed for co-localization of Fos and TPH2, we counted the number of single- and double-labeled cells in regions of every section containing TPH2-postive neurons (37–65 sections/animal, median of 48 sections/animal). For this analysis, the following divisions of the raphe nuclei were considered, as defined in a previous manuscript [84]: raphe obscurus, raphe pallidus, raphe magnus, and the medial and lateral regions of the dorsal raphe nucleus.

### Statistical Analysis

Principal component analysis, conducted using Systat 11 (Systat Software, Chicago, IL), was used to identify a set of statistically independent (orthogonal) principal components that are sufficient to explain the numbers of Fos labeled cells in 23 sampled nuclear or subnuclear groups. The analysis was performed on the correlation matrix. An equamax rotation was used as a compromise of varimax and quartimax criteria, which minimizes both the number of neural structures (variables) that load highly on a component (network) and the number of components (networks) needed to explain the behavior of a neural structure (variable). The standardized component scores were calculated for each subject.

## Results

Table 2 provides details about behaviors exhibited by the animals during the stimulation session. Throughout the period of galvanic vestibular stimulation, three of the animals (C39, C52, C15) displayed motor responses characteristic of activation of labyrinthine receptors (sinusoidal head movement at the frequency of the stimulus and nystagmus), as well as prodromal signs of vomiting (periods of retching, copious salivation, frequent licking, panting). At the end of the session, two of the three animals were found to have defecated in the restraint bag; the watery diarrhea was suggestive of an acute stress effect on gastrointestinal motility [86]. The cumulative behavioral score for these three animals ranged from 12–20 (see Table 2), and they were classified as having strong autonomic responses to labyrinthine stimulation (response type 1). Three other animals (C62, C64, C02) exhibited overt behaviors indicating that the vestibular system was activated by the stimulus, particularly sinusoidal head roll at the frequency of the stimulus, although only one or two potential indicators of motion sickness were evident for each animal (see Table 2). These three animals had behavioral scores of 5–7, and were classified as having response type 2. Two other animals (C20 and C41) had little motor response to galvanic vestibular stimulation, although both animals slept throughout most of the stimulation session; their somnolence could have been a consequence of the stimulus. These two animals were grouped with two control animals that were not stimulated as having response type 3. The cumulative behavioral scores for type 3 animals ranged from 0–4.

### Expression of Fos by Brainstem Neurons Following Galvanic Vestibular Stimulation

**Figure 1:**
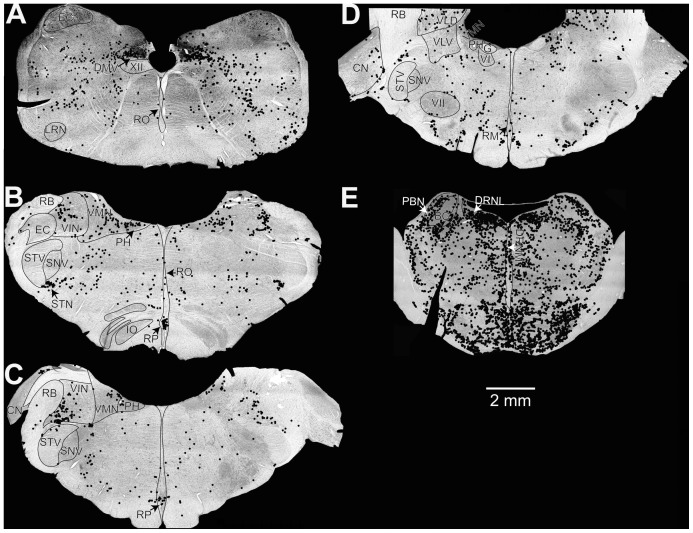
Locations of Fos-labeled neurons in two animals exhibiting strong symptoms of motion sickness (response type 1) during galvanic vestibular stimulation. Neuronal locations were plotted on photomontages of sections taken using a 4X objective. Sections (A, E) are from animal C52, whereas (B–D) are from animal C39. The sections were located at the following approximate distances posterior to stereotaxic zero, in accordance with Berman’s atlas [85]: A, 13.5 mm; B, 10 mm; C, 9 mm; D, 7 mm; E, 3 mm. *Abbreviations*: BC, brachium conjunctivum; CN, cochlear nuclei; DMV, dorsal motor nucleus of the vagus; DRNL, lateral division of dorsal raphe nucleus; DRNM, medial division of dorsal raphe nucleus; EC, external cuneate nucleus; G, genu of facial nerve; IO, inferior olivary nucleus; LRN, lateral reticular nucleus; PBN, parabrachial nucleus; PH, prepositus hypoglossi; RB, restiform body; RM, raphe magnus; RO, raphe obscurus; RP, raphe pallidus; SNV, spinal trigeminal nucleus; STN, subtrigeminal nucleus; STV, spinal trigeminal tract; VI, abducens nucleus; VII, facial nucleus; VIN, inferior vestibular nucleus; VLD, dorsal division of lateral vestibular nucleus; VLV, ventral division of lateral vestibular nucleus; VMN, medial vestibular nucleus; XII, hypoglossal nucleus.

**Figure 2:**
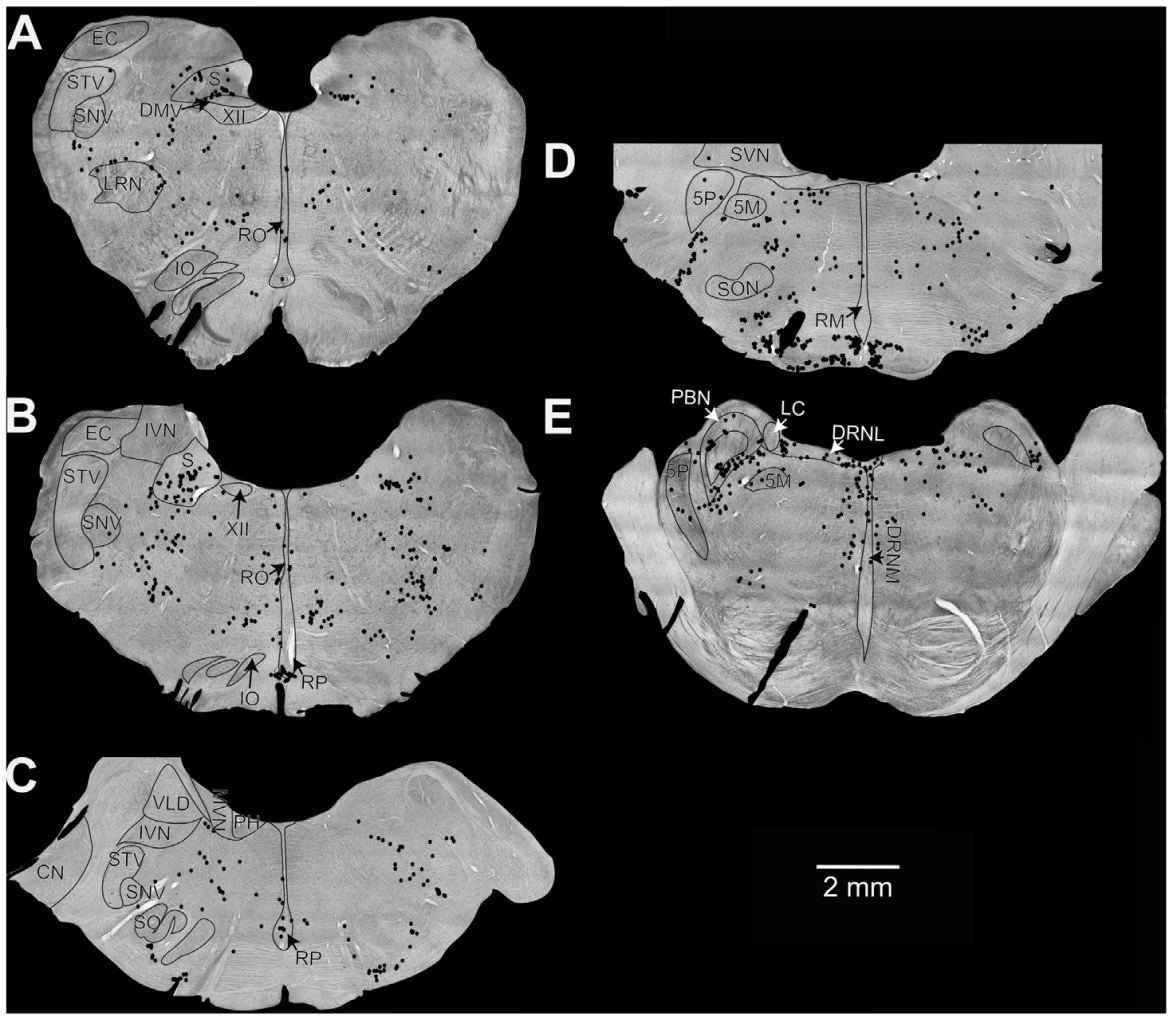
Locations of Fos-labeled neurons in the two unstimulated control animals (C83 and C84). Neuronal locations were plotted on photomontages of sections taken using a 4X objective. Sections (**A, B, E**) are from animal C83, whereas (**C, D**) are from animal C84. The sections were located at the following approximate distances posterior to stereotaxic zero, in accordance with Berman’s atlas: **A,** 13.5 mm; **B,** 12 mm; **C,** 8 mm; **D,** 6 mm; **E,** 4 mm. Abbreviations are the same as in Fig. 1, with the following additions: 5M, motor trigeminal nucleus; 5P, principal trigeminal nucleus; LC, locus coeruleus; S, solitary nucleus; SO, superior olivary nucleus; SVN, superior vestibular nucleus.

**Figure 3:**
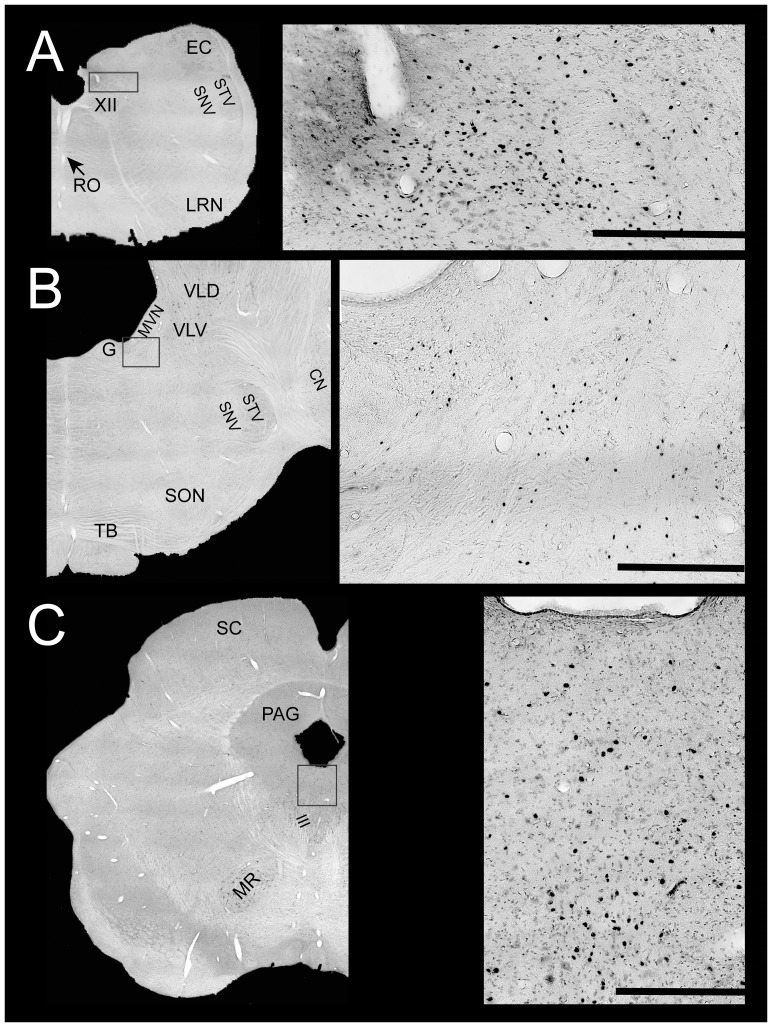
Photomicrographs of Fos-labeled neurons in a response type 1 animal (C52). In each row, a rectangular box on the left diagram (generated from photomontages of sections taken using a 4X objective) shows the region depicted at higher magnification to the right. Scale bars on the right photomicrographs designate 500 µm. **A,** Fos labeling in nucleus tractus solitarius, approximately 13.5 mm posterior to stereotaxic zero. **B,** Fos labeling in the rostral portion of the medial vestibular nucleus, approximately 6 mm posterior to stereotaxic zero. **C,** Fos labeling in the periaqueductal gray, approximately 3 mm rostral to stereotaxic zero. Abbreviations are the same as in Figs. 1–2, with the following additions: III, oculomotor nucleus; MR, magnocellular portion of the red nucleus; PAG, periaqueductal gray; SC, superior colliculus.

**Figure 4:**
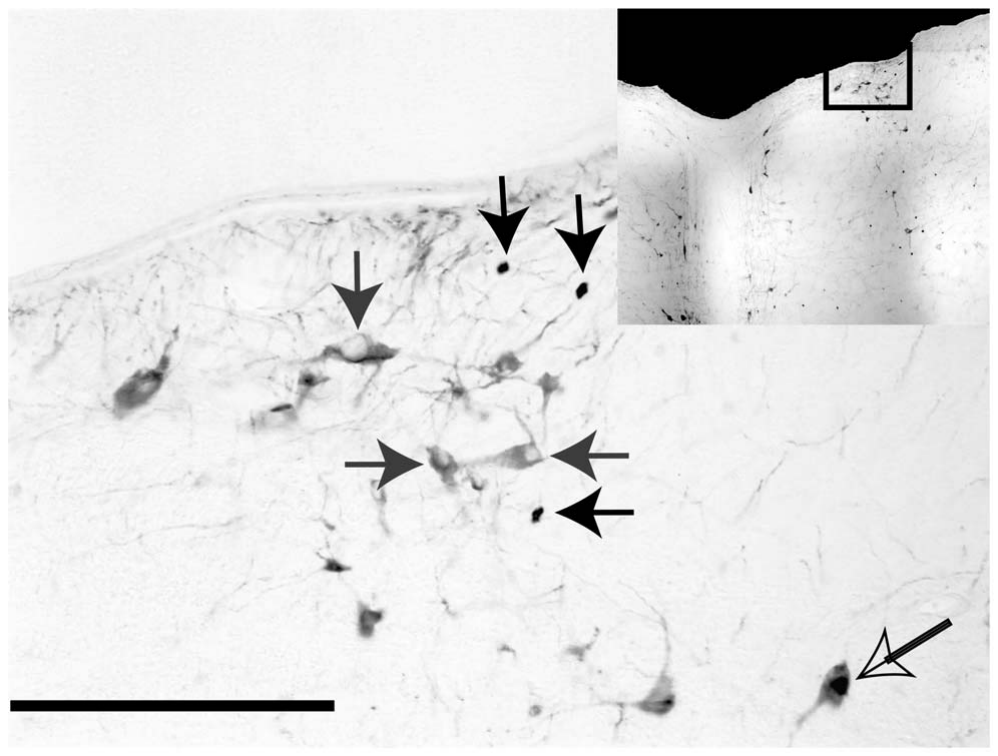
Photomicrograph illustrating examples of neurons that were immunopositive for Fos (solid black arrows), TPH-2 (solid gray arrows) and both TPH-2 and Fos (open arrow). A rectangular box on the inset diagram indicates the region of the dorsal raphe nucleus depicted in the photomicrograph. The scale bar represents 250 µm.

Maps of the locations of Fos-labeled neurons in animals with a strong autonomic response are provided in Fig. 1, whereas Fig. 2 shows the locations of Fos labeling in response type 3 animals. Photomicrographs of Fos-labeled neurons from a response type 1 animal are provided in Fig. 3. Fig. S1 also allows for a comparison of Fos labeling in several brainstem regions containing serotoninergic neurons in animal C62. Following a qualitative review of the sections, counts of the number of Fos-immunopositive neurons were obtained from the following areas having a high density of labeling in a subset of animals: inferior vestibular nucleus, medial vestibular nucleus (caudal and rostral aspects), lateral vestibular nucleus (dorsal and ventral divisions), superior vestibular nucleus (lateral and medial aspects), NTS (lateral, medial, and commissural nuclei), periaqueductal gray (dorsal, lateral, ventral, and ventrolateral divisions), parabrachial nucleus complex (medial and lateral parabrachial nuclei and the adjacent Kölliker-Fuse nucleus), locus coeruleus, the subtrigeminal nucleus (region ventral to the spinal trigeminal nucleus), the external cuneate nucleus, and the subdivisions of the raphe nuclei. Because there was no significant difference between the number of labeled cells in any structure on the left versus right side, the total number of labeled cells was used for principal component analysis. Since the raphe nuclei contained many Fos-immunopositive neurons in some animals, we performed dual-labeling immunohistochemistry on an additional well of tissue from all animals except the unstimulated controls to co-localize Fos and TPH2; examples of dual-labeled cells are illustrated in Fig. 4, as well as Fig. S1.

### Correlations between Fos Labeling and Symptoms

The numbers of Fos labeled neurons in a subset of the sampled brain regions showed a strong positive correlation with both the total symptom severity score and a subscore for autonomic manifestations of motion sickness (subscore for frequent licking, retching, excessive salivation, vocalization, panting, defecation during the stimulation session, urination during the stimulation session, and sedation [sleeping during the majority of the stimulation session]) (Table 3). The positive correlation was strong in the medial, lateral and inferior vestibular nuclei, but a weak negative correlation was present in the superior vestibular nucleus. The correlation was also strong in medial and lateral subnuclei of the solitary nucleus, but not significant in the commissural solitary nucleus. Strong positive correlations were also observed with Fos labeling in locus coeruleus, Kölliker-Fuse nucleus, non-serotonergic cells (i.e., cells that were not immunopositive for TPH2) in the nucleus raphe magnus and the medial and lateral parabrachial nuclei, but not in the serotonergic (TPH2-immunopositive) cells in the raphe nuclear groups, subtrigeminal nucleus, or any division of the periaqueductal gray. There was a weak negative correlation between the number of labeled cells and symptom severity in the dorsal raphe nucleus and the raphe pallidus et obscurus. Stepwise multiple regression (removal criterion p = 0.15) indicated that the motion sickness symptom score could be represented (adjusted multiple r-squared = 0.653) as a weighted sum of the number of labeled cells in lateral subnucleus of the nucleus of the solitary tract (coefficient: 0.123), subtrigeminal nucleus (coefficient: 0.143) and external cuneate nucleus (coefficient: 0.033) plus a constant term (−1.567). The subscores for autonomic manifestations of motion sickness showed a more robust regression result (adjusted multiple r-squared = 0.951) as a weighted sum of the number of labeled cells in the Kölliker-Fuse nucleus (coefficient: 0.119) and subtrigeminal nucleus (coefficient: −0.088) plus a constant term (−0.398).

**Table 3:** Correlation of labeling with symptom score and component loadings from principal component analysis with equamax rotation.

Structure	Correlation re: Signs,Total *(Autonomic only*)	Component 1	Component 2	Component 3	Component 4
Lateral Vestibular N.	**0.879 (0.886)**	**0.901**	0.305	0.074	0.297
Medial Vestibular N.	**0.715 (0.680)**	**0.926**	0.287	0.201	0.136
Locus Coeruleus	**0.874 (0.844)**	**0.861**	0.248	0.084	0.434
Periaqueductal N.	0.145 (0.056)	**0.856**	0.091	−*0.504*	−0.001
Nucleus Tractus Solitarius	0.647 (0.600)	**0.687**	**0.698**	0.134	0.001
Inferior Vestibular N.	**0.784 (0.789)**	**0.672**	**0.545**	0.076	0.493
Medial Parabrachial N.	**0.710 (0.702)**	**0.636**	0.440	−0.255	**0.580**
Kölliker-Fuse N.	**0.901 (0.947)**	**0.538**	**0.630**	0.309	0.447
Lateral Parabrachial N.	**0.785 (0.786)**	0.006	**0.967**	0.213	0.132
Dorsal Raphe N. (TPH2)	−0.395 (−0.430)	−0.359	−***0.612***	0.422	−***0.517***
Superior Vestibular N.	0.487 (0.476)	−0.068	−0.329	−***0.815***	−0.468
External Cuneate	0.392 (0.371)	−0.060	−0.260	−***0.951***	0.075
Subtrigeminal N.	0.450 (0.500)	−0.020	0.334	−***0.732***	0.468
Dorsal Raphe N. (non-TPH2)	−0.23 (−0.316)	−0.015	−0.029	0.007	−***0.990***
% Variance explained		34.99%	22.98%	20.15%	19.87%
r^2^ of Score re: Signs		0.41 (0.29)	0.26 (0.29)	0.26 (0.31)	0.05 (0.11)

**Bold type** is used to highlight regions with strong negative or positive correlations between the number of Fos-immunopositive neurons and symptom severity scores. **Bold type** also designates large positive component loadings; ***bold and italicized type*** designates negative component loadings. *TPH2: Tryptophan hydroxylase 2,* a marker of brain serotoninergic neurons.

### Principal Component Analysis: Identifying Connected Network-like Behavior from Fos Data

The range of behavioral responses provides an opportunity for identifying interconnected neural response networks from functional correlations between Fos labeling in different nuclei in the animals. The relationships between labeling across nuclei were identified with an approach from principal component analysis, a multivariate technique that was developed initially by Hotelling [87] to identify a smaller number of independent variables that can determine the values of observations from a larger number of correlated independent variables. From a practical perspective, it provides criteria to identify statistically independent, linear combinations (principal components) of the measured variables that explain the majority of the variance in the data [88]. Network contributions of different nuclei can then be identified by shared, high loadings on a particular component.

Results of principal component analysis of data from twelve nuclear groups, plus serotonergic and non-serotonergic dorsal raphe nucleus cells, are summarized in Table 3. The Kaiser-Meyer-Olkin statistic of 0.74 and significant Bartlett’s test for sphericity (p<0.05) indicated that the sample was adequate for the principal component analysis [89]. Four orthogonal principal components (eigenvalues: 8.062, 2.924, 1.724, and 1.009) were identified. These statistically independent components are expressed as component loadings for each of the brain regions; the loadings represent the relationship (on a range between −1 and 1) between the component and the normalized (z-transformed) labeling in that region. The polarities are arbitrary; opposite polarities indicate a ‘push-pull’ relationship. For a given factor, large magnitude loadings for different nuclei represent the strength of the linear relationship of Fos labeling to that component. If a group of structures is part of a known connected pathway, then large magnitude loadings across those structures are consistent with engagement of that network.

Component 1 (explains 34.99% of the variance) had large positive loadings for the inferior vestibular nucleus, medial vestibular nucleus, lateral vestibular nucleus, periaqueductal gray, solitary nucleus, locus coeruleus and medial parabrachial nucleus, and a weaker positive loading from the Kölliker-Fuse nucleus. Component 2 included large positive contributions from the solitary nucleus, inferior vestibular nucleus, Kölliker-Fuse nucleus, and lateral parabrachial nucleus, with an opposite polarity contribution from serotonergic dorsal raphe cells. Component 3 had large contributions from the superior vestibular nucleus, periaqueductal gray, external cuneate nucleus and the subtrigeminal nucleus. Finally, Component 4 had a strong contribution from non-serotonergic dorsal raphe neurons, with a lesser contribution from serotonergic dorsal raphe cells and a contribution of opposite polarity from the medial PBN. A principal components analysis that included nuclear subdivision and other raphe nuclei (Table 4) indicated that five principal components (eigenvalues of 12.263, 6.011, 3.81, 2.61 and 1.307) could account for greater than 95% of the variance in Fos labeling. Four of the components correspond to the results of the analysis in Table 3 and have been named identically.

**Table 4:** Correlation of labeling with symptom score and component loadings from principal component analysis with equamax rotation, with nuclear subdivisions included.

Structure	Correlation re:Symptoms, Total(Autonomic only)	Component 1	Component 2	Component 2a	Component 3	Component 4
Dorsal Lateral Vestibular N.	**0.742 (0.757)**	**0.934**	0.184	0.12	0.261	−0.108
Rostral Medial Vestibular N.	**0.726 (0.673)**	**0.929**	0.176	0.269	0.154	−0.099
Caudal Medial Vestibular N.	**0.687 (0.662)**	**0.872**	0.346	0.321	−0.081	0.096
Locus Coeruleus	**0.874 (0.844)**	**0.85**	0.292	0.25	0.135	0.334
Lateral Periaqueductal Gray	0.202 (0.120)	**0.837**	0.019	−0.095	**0.537**	0.031
Ventral Lateral Vestibular N.	**0.780 (0.773)**	**0.826**	0.361	0.321	0.087	0.278
Lateral Nucleus Tractus Solitarius	**0.652 (0.601)**	**0.764**	**0.55**	0.16	−0.203	0.219
Dorsal Periaqueductal Gray	0.082 (−0.009)	**0.734**	0.048	−0.022	0.102	***−0.67***
Ventrolateral Periaqueductal Gray	0.115 (0.074)	**0.738**	0.101	0.127	0.65	0.083
Inferior Vestibular N.	**0.784 (0.789)**	**0.635**	**0.527**	0.398	0.241	0.321
Ventral Periaqueductal Gray	0.079 (−0.129)	**0.614**	0.007	−0.409	**0.674**	−0.034
Medial Parabrachial N.	**0.710 (0.702)**	**0.606**	0.428	0.185	**0.509**	0.395
Kölliker−Fuse N.	**0.901 (0.947)**	0.491	**0.584**	**0.58**	0.067	0.276
Lateral Parabrachial N.	**0.785 (0.786)**	−0.056	**0.929**	0.363	0.039	0.005
Raphe Magnus (non-TPH2)	0.492 (0.467)	0.171	**0.894**	0.3	−0.253	0.137
Medial Nucleus Tractus Solitarius	**0.652 (0.635)**	0.27	**0.873**	0.128	0.273	0.271
Raphe Magnus (TPH2)	0.096 (0.066)	−0.167	**0.751**	0.28	−0.079	−***0.569***
Dorsal Raphe Nucleus (TPH2)	−0.395 (−0.43)	−0.331	−***0.641***	0.049	−***0.585***	−0.367
Raphe Pallidus/Obscurus (TPH2)	−0.359 (−0.487)	0.085	−0.262	−***0.951***	−0.102	0.097
Raphe Pallidus/Obscurus (non−TPH2)	−0.450 (−0.538)	−0.432	0.081	−***0.753***	0.489	0.019
Medial Superior Vestibular N.	**0.502 (0.495)**	−0.029	−0.428	−***0.689***	0.108	−***0.575***
External Cuneate	0.392 (0.371)	−0.044	−0.266	−***0.611***	**0.744**	0.029
Lateral Superior Vestibular N.	0.454 (0.442)	−0.079	−0.253	−***0.545***	**0.733**	−0.308
Subtrigeminal N.	**0.450 (0.500)**	−0.077	0.118	0.157	**0.964**	0.161
Commissural Nucleus Tractus Solitarius	−0.051 (−0.100)	0.062	−0.037	0.07	0.075	−***0.992***
Dorsal Raphe N. (non-TPH2)	−0.230 (−0.316)	−0.042	−0.104	−0.15	−0.25	−***0.95***

**Bold type** is used to highlight regions with strong negative or positive correlations between the number of Fos-immunopositive neurons and symptom severity scores. **Bold type** also designates large positive component loadings; ***bold and italicized type*** designates negative component loadings. *TPH2: Tryptophan hydroxylase 2,* a marker of brain serotoninergic neurons.

The first component (Component 1 in Table 4) corresponds to an anatomical refinement of Component 1 from the initial analysis (Table 3). Large positive loadings were observed for most vestibular nucleus divisions (except the superior vestibular nucleus), the lateral subnucleus of the solitary nucleus, medial parabrachial and Kölliker-Fuse nuclei and (in decreasing order) the dorsal, lateral, ventrolateral and ventral divisions of the periaqueductal gray. It also has very high loading for the locus coeruleus, but very low loadings for the dorsal raphe and nucleus raphe magnus. It accounts for 31.2% of the between-animal variance in the data. The normalized component scores showed a positive correlation (r^2^ = 0.387) with the cumulative behavioral scores and with the cumulative autonomic subscores (r^2^ = 0.53).

Component 2 in Table 4 corresponds to a refinement of Component 2 from the initial analysis presented in Table 3. It reflects push-pull interactions between two subnetworks. One subnetwork reflects strong factor loadings with Fos expression in the lateral parabrachial nucleus, medial subnucleus of the solitary tract, and nucleus raphe magnus (both serotonergic and non-serotonergic cells) as well as more moderate loadings from the Kölliker-Fuse nucleus, inferior vestibular nucleus, and lateral subnucleus of the solitary tract. A contribution of opposite polarity comes from serotonergic cells in the dorsal raphe nucleus. Component 2 accounts for 20.5% of the between-animal variance in the data. The normalized component scores were uncorrelated (r^2^ = 0.141) with the cumulative behavioral scores and with the autonomic subscores (r^2^ = 0.018).

Component 2a reflects a moderate positive loading from the Kölliker-Fuse nucleus, with contributions of opposite polarity from a network that includes strong influences of the serotonergic and non-serotonergic cells in the nuclei raphe pallidus et obscurus and the medial aspect of the superior vestibular nucleus, with more moderate contributions from the lateral aspect of the superior vestibular nucleus and external cuneate nucleus. It accounts for 15.7% of the variance. The normalized component scores were uncorrelated (r^2^ = 0.04) with the cumulative behavioral scores and with the autonomic subscores (r^2^ = 0.002). It appears to have been embedded in components 2–4 of the initial analysis, primarily by the pooling of the medial and lateral superior vestibular nucleus.

Component 3 in Table 4 corresponds to an anatomical refinement of component 3 from the initial analysis. It has large positive loadings for the subtrigeminal nucleus, lateral aspect of the superior vestibular nucleus, external cuneate nucleus, and the ventral and ventrolateral divisions of the periaqueductal gray, and moderate loadings from the medial parabrachial nucleus, lateral subnucleus of the periaqueductal gray and non-serotonergic neurons in nuclei raphe pallidus et obscurus. The serotonergic dorsal raphe neurons have a relatively strong loading of opposite polarity. Factor 4 accounts for 17.3% of the between-animal variance. Normalized component scores showed a positive correlation (r^2^ = 0.35) with the cumulative behavioral scores and with the autonomic subscores (r^2^ = 0.325).

Finally, Component 4 in Table 4, an anatomical refinement of Component 4 of the initial analysis, has high loadings (negative polarity) for non-serotonergic cells in the dorsal raphe nucleus, commissural subnucleus of the solitary nucleus, and the dorsal region of the periaqueductal gray, with more moderate loadings for the medial aspect of the superior vestibular nucleus (the terminus of periventricular plexus (small caliber) non-serotonergic dorsal raphe axons) and serotonergic cells in nucleus raphe magnus. Component 5 accounts for 15.2% of the between-animal variance. The normalized component scores among animals were uncorrelated (r^2^ = 0.080) with the cumulative behavioral scores and with the autonomic subscores (r^2^ = 0.125).

## Discussion

The nuclear expression of the early immediate gene transcription factor Fos has been used for several decades to identify neurons affected by a variety of peripheral and central stimuli [29], [90]. For example, Fos expression patterns can be used as a marker of anatomically and physiologically identified pain and autonomic pathways in different contexts [91]–[95]. The brain stem structures that were selected for Fos quantification have been implicated in either *(1)* vestibular contributions to autonomic, affective and somatic pathways [96], [97], *(2)* Fos activation by vestibular stimulation [98] or *(3)* generation of nausea, emesis and responses to noxious stimulation [22], [27], [91], [92], and include both the raphe nuclei and locus coeruleus. These structures have also shown Fos activation after both natural vestibular stimulation [99], [100] and acute unilateral vestibular damage [101].

Principal component analysis is an exploratory multivariate statistical approach to identify a reduced set of orthogonal principal components that account for the variability in a larger data set of many measured variables [88], [102]. This approach was pioneered in the early 1930s by Hotelling [87]. From a mathematical perspective, principal components are the characteristic vectors of the covariance or the correlation matrix of a data set. Principal components are linear combinations of measured variables that have large variances and, therefore, can account for the variation in the data in terms of positive and negative interactions among the measurements. Hence, the approach is suited well for identifying presumptive interactions between groups of neurons (measured variables such as cFos labeling) contributing to functional pathways (principal components).

The experiment was designed explicitly to facilitate the use of principal component analysis for identifying functional pathways on the basis of synchronous Fos expression during the generation of behaviors culminating in motion sickness. Firstly, existing knowledge regarding vestibular and autonomic pathways permits ‘sense-making’ by associating the identified principal components with neuronal networks. By sampling cFos labeling from nuclei that contribute to known networks for processing vestibular and autonomic information, the contributions of neuronal populations to individual principal components can be interpreted in terms of network activity. Secondly, because the data set consisted of individual experimental animals displaying different degrees (manifestations) of motion sickness, the components across the population provide an estimate of activity in the associated networks across the behavioral response spectrum. Hence, the approach exploits the individual variation in the severity of behavioral motion sickness to identify co-modulated neuronal pathways as principal components that explain underlying variance in the Fos labeling. It is analogous to strategies for identifying synchronous, connected networks from electrophysiological [103] or functional imaging data [104]. Therefore, the five components identified by this approach represent coordinated engagement of pathways along the progression from mild discomfort to emesis.

Components 1 and 4 showed a positive correlation with the cumulative behavioral scores, especially with the autonomic subscore. Hence, they are likely related to the overt signs of motion sickness reflected in the behaviors monitored in this study. Component 1 reflects correlates of changes in vestibular nucleus Fos activation during galvanic vestibular stimulation. The factor is anchored by the strong covariance in Fos activation within locus coeruleus, vestibular nuclei, lateral NTS, medial parabrachial nucleus and periaqueductal gray, and a more moderate relationship with the Kölliker-Fuse nucleus. Because anatomical studies have documented direct, strong interconnections among these structures, the Fos co-regulation represents coordinated engagement of this network across animals with different levels of behavioral responses to galvanic (or sham) stimulation. Locus coeruleus receives direct projections from the vestibular nuclei [105], [106] and its neurons respond to both vestibular and neck stimulation [107]. Locus coeruleus also contributes a regionally specialized projection to the vestibular nuclei [108]. The medial parabrachial nucleus is connected reciprocally with the vestibular nuclei [105], [109]; the lateral NTS receives direct input from the vestibular nuclei [51], [110] and projects to the external medial parabrachial and Kölliker-Fuse nuclei [111]. The periaqueductal gray receives light projections from the vestibular nuclei [112], is connected reciprocally with the locus coeruleus [113], [114], and projects to the medial and lateral parabrachial nuclei [115]. Recent studies in rats indicate that Fos activation in locus coeruleus and the periaqueductal gray is observed in animals displaying escape responses in an elevated T-maze task [116], which may include recruitment of anxiolytic effects of norepinephrine via the dorsal periaqueductal gray [117].

Component 3, like Component 1, identifies correlated Fos labeling in a network involving interconnections between the periaqueductal gray and medial parabrachial nucleus [115]. However, unlike Component 1, it has negligible association with Fos labeling in the lateral, medial and inferior vestibular nuclei. Rather, the labeling is associated with Fos activation in the lateral aspect of the superior vestibular nucleus and two precerebellar regions that are activated by prolonged, natural linear acceleration (otolith organ) stimuli, the subtrigeminal and external cuneate nuclei [98]. The positive association of Fos labeling in non-serotonergic raphe pallidus et obscurus neurons is also of opposite polarity to the contribution to Component 1, and the serotonergic dorsal raphe labeling has a loading similar to Component 2. Thus, this Fos activation may reflect the fact that the lateral aspect of the superior vestibular nucleus receives large caliber serotonergic dorsal raphe nucleus projections, but negligible non-serotonergic inputs from the dorsal raphe nucleus via periventricular plexus [118].

A Fos coactivation of cells in the lateral periaqueductal gray and dorsal raphe nucleus was noted recently in association with avoidance responses to an elevated T-maze task [116] and during defensive behaviors in rodents [119]. Finally, the correlated periaqueductal gray and subtrigeminal nucleus contributions are of interest in light of similar activation in imaging studies during offset analgesia [120]. We suggest that this network is reflects responses to aversive aspects of visuospatial discomfort and anxiety.

Components 2, 2a, and 4 were uncorrelated with the cumulative behavioral scores, and thus were not related to the overt signs of motion sickness. Instead, these factors were likely related to the covert symptoms of motion sickness that are not readily evident (e.g., affective components of the condition). Component 2 reflects interactions of opposite polarity from two interconnected networks related to nucleus raphe magnus and the serotonergic cells in the dorsal raphe nucleus, respectively. Strong positive factor loadings for Fos labeling were identified in a network of structures that are interconnected with serotonergic and non-serotonergic cells of nucleus raphe magnus [121]–[125], including strong loadings for the lateral parabrachial nucleus and medial subnucleus of the solitary tract and more moderate, positive loadings for labeling in the medial parabrachial nucleus, Kölliker-Fuse nucleus, inferior vestibular nucleus, and lateral subnucleus of the solitary tract. This Fos activity likely reflects interconnections between the inferior vestibular nucleus, Kolliker-Fuse nucleus and nuclei of the solitary tract [51], [105], [109], [111], [126]. These interconnections are likely involved in generation of discharges of serotonergic nucleus raphe magnus neurons in association with autonomic and respiratory activity [127]. Contributions of opposite polarity are provided by Fos labeling of serotonergic cells in the dorsal raphe nucleus, and, to a more moderate extent, from the medial aspect of the superior vestibular nucleus, which receives serotonergic input from the dorsal raphe nucleus [118]. Because several structures had significant positive loadings on this component, and nucleus raphe magnus and the medial and lateral subnuclei of the solitary nucleus provide inputs to the dorsal raphe nucleus [128], this component suggests a prominent push-pull interplay between raphe magnus and dorsal raphe circuits in the development of autonomic and respiratory components of nausea and emesis. More significantly, they likely reflect the role of nucleus raphe magnus neurons in descending modulation of sensitivity to aversive visceral and somatic sensory information [129], analogous to its role in maintenance of hyperalgesia and allodynia after nerve injury and inflammation [130].

Component 2a reflects an opposite relationship between Fos activation in the Kölliker-Fuse nucleus and concurrent Fos inhibition in a network that includes serotonergic and non-serotonergic cells in the nuclei raphe pallidus et obscurus, neurons in the superior vestibular nucleus and external cuneate nucleus. Although Fos labeling in each of the latter structures is correlated negatively with symptom score (Table 4), the factor scores were uncorrelated with the magnitude of the symptom score. The relationships between Fos labeling in the caudal raphe nuclei, superior vestibular nucleus and Kölliker-Fuse nucleus in Component 2a are statistically independent of the relationships between the latter structures and more rostral raphe nuclei in Component 2, which is suggestive of different functions. Both the Kölliker-Fuse and superior vestibular nuclei project to raphe pallidus et obscurus [122] and these caudal raphe nuclei contribute serotonergic and non-serotonergic projections to the vestibular nuclei [131]. A recent optogenetic study [132] reported that activation of serotonergic neurons in raphe obscurus increased respiratory amplitude and frequency as well as the sensitivity of central respiratory chemoreflexes. The caudal raphe nuclei have also been implicated as a modulator of other somatosympathetic responses, including cardiovascular sympathoexcitatory reflexes during acupuncture [133]. Hence, the network contributing to this factor may be modulating respiratory and autonomic motor activity associated with the magnitude of development of motion sickness signs.

Component 5 is anchored by high magnitude contributions from Fos labeling in the commissural nucleus tractus solitarii and non-serotonergic dorsal raphe neurons and moderate loadings from the dorsal periaqueductal gray, serotonergic neurons in the nucleus raphe magnus and the medial aspect of the superior vestibular nucleus. This activity may reflect a network anchored by commissural nucleus tractus solitarii projections to the dorsal raphe nucleus [128] and non-serotonergic dorsal raphe projections to the medial aspect of the superior vestibular nucleus [118]. The serotonergic raphe magnus contribution may also reflect its afferent relations with the periaqueductal gray [134] and strong projections to nucleus tractus solitarii [123]. Because activation the dorsal periaqueductal gray contributes to aversion and anxiety-like responses [135], we suggest this network is involved in generation of integrated vestibular-visceral aversive signals related to motion sickness.

The principal component analysis approach also highlighted several striking global relationships for Fos activation patterns in the locus coeruleus (noradrenergic), and the serotonergic and non-serotonergic cell groups in the dorsal raphe nucleus, nucleus raphe magnus and nuclei raphe pallidus et obscurus. Specifically, different combinations of these groups were associated with different statistically independent components. Firstly, locus coeruleus Fos labeling patterns are independent statistically from Fos labeling of serotonergic neurons in the dorsal raphe, raphe magnus and raphe pallidus et obscurus. Secondly, the Fos labeling of serotonergic and non-serotonergic dorsal raphe nucleus cells tend to behave independently, which suggests that their differential projections to the vestibular nuclei and ascending pathways [118] are associated with different roles in responses to galvanic stimulation leading to autonomic arousal. Thirdly, reciprocal (push-pull) relationships between these different cell groups were prominent in three of the factors: locus coeruleus versus nuclei raphe pallidus et obscurus for Component 1, serotonergic and non-serotonergic nucleus raphe magnus neurons versus serotonergic dorsal raphe nucleus neurons for Component 2, and serotonergic dorsal raphe neurons versus non-serotonergic raphe pallidus et obscurus neurons on Component 4. Finally, Component 5 featured parallel Fos labeling of serotonergic nucleus raphe magnus and non-serotonergic dorsal raphe nucleus neurons.

## Summary and Conclusions

The identification of five statistically independent component networks associated with the development of motion sickness provides an opportunity to consider, in network activation dimensions, the complex progression of signs and symptoms that are precipitated in provocative environments (both real and virtual). The inability of numerous studies to identify autonomic pathognomonic patterns during the development of motion sickness [136] leave us to rely on subjective symptoms and symptom clusters. Hence, it will be important to determine the relationships between temporal patterns of activity in these networks and the behavioral and perceptual dimensions used to assess the severity of motion sickness. In essence, this approach may allow us to formulate motion sickness in terms of General Recognition Theory [137], by framing the component networks as orthogonal dimensions (processes) that produce progressive, perceptually independent clusters of signs and symptoms.

Schemata for the dimensions underlying subjective signs and symptoms of motion sickness have been derived from multivariate analysis of responses to questionnaires [138], [139]. For example, the Simulator Sickness Questionnaire (SSQ) scoring metric was constructed from a principal component analysis of the original Pensacola Motion Sickness Questionnaire [138]. The authors identified orthogonal dimensions that they termed nausea (and/or gastrointestinal awareness), vision and visuomotor (eyestrain) function, and disorientation (dizziness, blurred vision and difficulty focusing) which have been used to document psychophysical differences in responses to different visual environments, simulator types, and stimulus patterns in vection drums [140]. A more recent Motion Sickness Assessment Questionnaire (MSAQ) is based upon four factors identified from a broader, two phase exploratory analysis [141]. The MSAQ gastrointestinal factor spans sensations from queasiness to nausea and vomiting. The MSAQ central factor includes dizziness, lightheadedness, disorientation and blurred vision. The MSAQ peripheral factor includes reports associated with autonomic responses such as sweatiness, clamminess, and hot/warm sensations. Finally, the MSAQ sopite-like factor captures diffuse fatigue, affective and emotional components associated with the ‘sopite syndrome’ [142], [143]. Because these questionnaire-based factors are only clusters of correlated symptoms, their relationship to metrics of activation of the networks identified in this study (e.g., from functional imaging studies) has the potential to bring etiologic precision to the diagnosis of prodromal trajectories of motion sickness.

The networks identified in this study include component pathways that have been described in the pain literature as subserving the affective dimension(s) of pain [144], particularly interoception and generation of feelings and emotions associated with activity in nociceptive afferent pathways [145]–[147]. In a broader sense, this overlap of networks for a pain and motion sickness is consistent with their role in core circuitry for determining the aversiveness of sensory patterns [148]. These networks also are likely contributors to the affective and emotional process of ‘being in pain’, which, as noted in 1968 by Melzack and Casey [149] and elaborated later by Grahek [150], can be dissociated from pain sensation and perception. Psychophysical studies have attempted to separate pain sensation and perception from pain aversiveness by asking subjects to rate both intensity and unpleasantness. It is of interest that for visceral and somatic pain stimuli of equal estimated intensity, the visceral pain is judged to be significantly more unpleasant [151]. Hence, activity in the five networks identified in our study may contribute to the aversive aspects of the development of motion sickness.

## Supporting Information

Figure S1 Plate comparing TPH2 and Fos labeling in three brain areas of animal C62: Dorsal raphe nucleus (A), Raphe magnus (B), and raphe pallidus (C). The calibration bar in this plate represents 500 µA in A and 250 µA in B–C.
(TIF)
