# Supplementary figures and images for: Identification of Neural Networks That Contribute to Motion Sickness through Principal Components Analysis of Fos Labeling Induced by Galvanic Vestibular Stimulation
Source: PLoS One. 2014 Jan 23;9(1):e86730. doi: 10.1371/journal.pone.0086730 (PMC3900607; doi:10.1371/journal.pone.0086730)

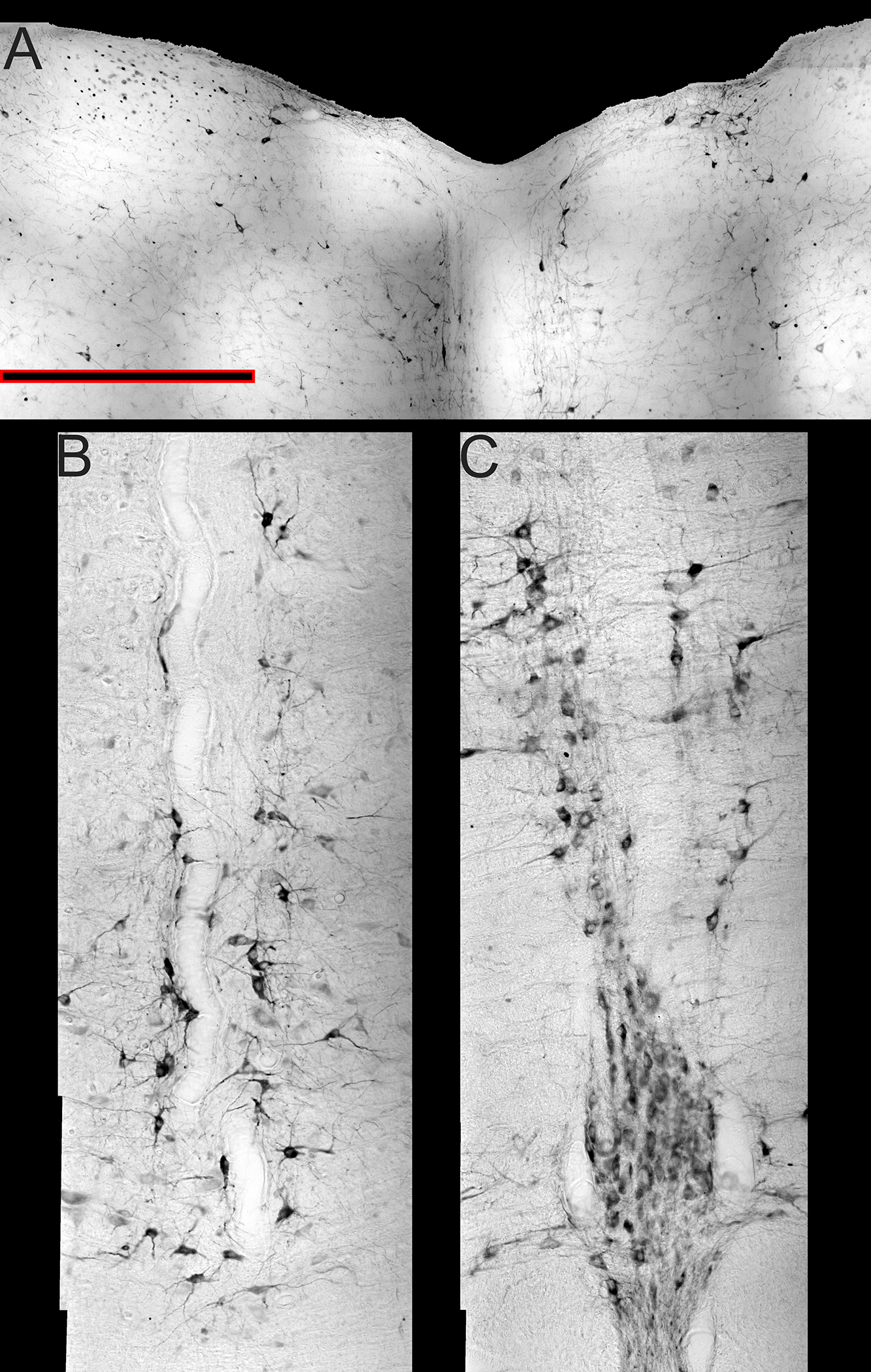

Supplement: Figure S1 — Plate comparing TPH2 and Fos labeling in three brain areas of animal C62: Dorsal raphe nucleus (A), Raphe magnus (B), and raphe pallidus (C). The calibration bar in this plate represents 500 µA in A and 250 µA in B–C. (TIF) [file pone.0086730.s001.tif]
